# Clinical Practice Guidelines on Ordering Echocardiography Before Hip Fracture Repair Perform Differently from One Another

**DOI:** 10.1007/s11420-020-09762-8

**Published:** 2020-06-08

**Authors:** Eric Swart, Chris Adair, Rachel B. Seymour, Madhav A. Karunakar

**Affiliations:** 1 Department of Orthopedics and Physical Rehabilitation, University of Massachusetts, Worcester, MA USA; 2 Department of Orthopedic Surgery, Atrium Health Musculoskeletal Institute, 1025 Morehead Medical Plaza, Suite 300, Charlotte, NC 28204 USA

**Keywords:** clinical practice guidelines, echocardiogram, pre-operative clearance, peri-operative management

## Abstract

**Background:**

Osteoporotic hip fractures typically occur in frail elderly patients with multiple comorbidities, and repair of the fracture within 48 h is recommended. Pre-operative evaluation sometimes involves transthoracic echocardiography (TTE) to screen for heart disease that would alter peri-operative management, yet TTE can delay surgery and is resource intensive. Evidence suggests that the use of clinical practice guidelines (CPGs) can improve care. It is unclear which guidelines are most useful in hip fracture patients.

**Questions/Purposes:**

We sought to evaluate the performance of the five commonly used CPGs in determining which patients with acute fragility hip fracture require TTE and to identify common features among high-performing CPGs that could be incorporated into care pathways.

**Patients and Methods:**

We performed a retrospective study of medical records taken from an institutional database of osteoporotic hip fracture patients to identify those who underwent pre-operative TTE. History and physical examination findings were recorded; listed indications for TTE were compared against those given in five commonly used CPGs: those from the American College of Cardiology/American Heart Association (ACC/AHA), the British Society of Echocardiography (BSE), the European Society of Cardiology and the European Society of Anaesthesiology(ESC/ESA), the Association of Anaesthetists of Great Britain and Ireland (AAGBI), and the Scottish Intercollegiate Guidelines Network (SIGN). We then calculated the performance (sensitivity and specificity) of the CPGs in identifying patients with TTE results that had the potential to change peri-operative management.

**Results:**

We identified 100 patients who underwent pre-operative TTE. Among those, the patients met criteria for TTE 32 to 66% of the time, depending on the CPG used. In 14% of those receiving TTE, the test revealed new information with the potential to change management. The sensitivity of the CPGs ranged from 71% (ESC/ESA and AAGBI) to 100% (ACC/AHA and SIGN). The CPGs’ specificity ranged from 37% (BSE) to 74% (ESC/ESA). The more sensitive guidelines focused on a change in clinical status in patients with known disease or clinical concern regarding new-onset disease.

**Conclusions:**

In patients requiring fixation of osteoporotic hip fractures, TTE can be useful for identifying pathologies that could directly change peri-operative management. Our data suggest that established CPGs can be safely used to identify which patients should undergo pre-operative TTE with low risk of missed pathology.

**Electronic supplementary material:**

The online version of this article (10.1007/s11420-020-09762-8) contains supplementary material, which is available to authorized users.

## Introduction

Osteoporotic fragility fracture of the hip is common and places significant clinical and financial burdens on the healthcare system [10, 11]. It typically occurs in elderly patients, many of whom are medically frail [17, 28] and have chronic medical issues that may not be controlled at the time of fracture [7, 17, 27, 28]. Because of these medical complexities, several clinical practice guidelines (CPGs) have been developed that make recommendations on the optimal management of such cases [1, 4, 5, 14, 15, 22].

The process of best preparing these medically complex patients for surgery is challenging. Typically, guidelines recommend rapid evaluation and surgical fixation of the fracture—within 24 to 48 h—with pre-operative workup and treatment limited only to things likely to directly affect peri-operative management [18, 20, 21, 23, 24, 26]. One evaluation that may delay surgical fixation, however, is transthoracic echocardiography (TTE) [6, 12, 16], which is used to assess for possibly treatment-limiting heart disease; requires a dedicated technician, not to mention the necessary equipment; and is not typically performed outside of daytime hospital hours [3, 13, 19, 25].

Studies have shown CPGs to be useful in determining who should undergo TTE [2, 8, 9], although criteria to determine who should receive TTE have not been firmly established, and several different CPGs have been proposed. Broadly, they tend to focus on the known or suspected presence of valvular disease, heart failure, or pulmonary hypertension, but they differ slightly in terms of criteria for screening with TTE. Some of these CPGs were specifically written with hip fracture patients in mind, whereas others were designed for use before elective, planned surgery. However, their accuracy in identifying which patients are likely to have pathology that necessitates TTE is not well established.

The purposes of this study were to evaluate the performance of five commonly used CPGs in determining which patients with acute fragility hip fractures should undergo TTE and to identify common features among high-performing CPGs that could be incorporated into care pathways.

## Methods

This was a retrospective cohort study of patients with osteoporotic hip fractures who underwent TTE as part of their pre-operative “clearance” evaluation. Patients were identified from a prospectively maintained database of hip fracture patients older than 55 years that included a record of who underwent TTE during hospitalization.

We received approval from the institutional review board at Atrium Health Musculoskeletal Institute in Charlotte, NC, USA, where the patients were treated. Study data were retrieved through a review of patient charts. Data extracted included pre-operative demographics and medical characteristics, including age, sex, comorbidities and medical history, and critical physical examination findings as documented in consultation notes from the medical and orthopedic teams. We also reviewed indications for TTE listed in the order, when available.

We then reviewed the patients’ medical histories and physical examination findings against the indications for TTE as specified in five commonly used CPGs: those from the American College of Cardiology/American Heart Association (ACC/AHA) [14], the British Society of Echocardiography (BSE) [5], the European Society of Cardiology and the European Society of Anaesthesiology (ESC/ESA) [15], the Association of Anaesthetists of Great Britain and Ireland (AAGBI) [1], and the Scottish Intercollegiate Guidelines Network (SIGN) [22] (Table 1).

**Table 1:** Indications for TTE, by guideline

Guideline	Indications for TTE
ACC/AHA [14]	• Dyspnea of unknown origin • Worsening of known heart failure signs or symptoms • Known history of valvular dysfunction or heart failure without echocardiography in last year or worsened symptoms • Suspicion of moderate or greater valvular stenosis or regurgitation
BSE [5]	• Documented ischemic heart disease • Unexplained dyspnea • Murmur with concomitant cardiac or respiratory symptoms • Murmur in asymptomatic patient where structural heart disease is suspected
ESC/ESA [15]	• Presumed or confirmed severe valvular disease
AAGBI [1]	• Dyspnea at rest or low level of exertion • Murmur suggestive of significant aortic stenosis
SIGN [22]	• New murmur that raises concerns about aortic stenosis • Known murmur in the presence of worsening clinical symptoms

*TTE* transthoracic echocardiography, *ACC/AHA* American College of Cardiology/American Heart Association, *BSE* British Society of Echocardiography, *ESC/ESA* European Society of Cardiology and the European Society of Anaesthesiology, *AAGBI* Association of Anaesthetists of Great Britain and Ireland, *SIGN* Scottish Intercollegiate Guidelines Network

We then reviewed the individual TTE reports for the presence of pathologies likely to change peri-operative medical management. The TTE reports for each patient were reviewed by a research team member blinded to CPG adherence. Indications for changes in management included a diminished left ventricular ejection fraction (below 25%); pulmonary hypertension, as indicated by an elevated right ventricular systolic pressure (greater than 55 mmHg); and the identification of new or worsening valvular disease classified as “moderate” or “severe” [6, 12]. Typical changes to management included the choice of anesthetic agent (e.g., spinal versus general anesthesia), peri-operative monitoring decisions (such as an arterial line or telemetry), and peri-operative fluid management.

Once we had determined which patients had an indication for changes to peri-operative management that included the use of TTE—according to each specific CPG—and those who underwent TTE that provided information that could alter medical management, we were able to determine the test-performance characteristics. We calculated sensitivity and specificity, comparing the test result (whether or not TTE was indicated according to a CPG) with the presence of actual disease found on TTE (i.e., a finding that could change peri-operative management, although we could not assess whether management did change).

## Results

Over a 4-year period, there were 538 patients over the age of 55 years presenting with osteoporotic fragility hip fractures. Of those, TTE was performed in 121 (22%). Of the patients in whom TTE was performed, 21 patients underwent it for a reason other than a finding from pre-operative evaluation (e.g., as part of a resuscitation code or an acute stroke evaluation), leaving 100 patients who met study criteria and were included in our analysis. The mean age at the time of admission was 82 years (range, 59 to 100 years; standard deviation, 10.5 years), and 74% of the patients were female. The most common comorbidities were hypertension (62%), congestive heart failure (42%), hyperlipidemia (29%), and diabetes mellitus (27%) (Table 2).

**Table 2:** Prevalence of comorbidities on presentation

Documented comorbidity on presentation	Prevalence
Hypertension	62%
Congestive heart failure	42%
Hyperlipidemia	29%
Diabetes mellitus	27%
Osteoporosis	24%
History of cancer	22%
Depression	22%
Chronic obstructive pulmonary disease	21%
Cerebrovascular accident	19%
Gastroesophageal reflux disease	17%
End-stage renal disease	4%
History of previous fragility fracture	4%
Hemodialysis	3%

Review of TTE reports showed that information with the potential to change management was identified in 14 patients (14%). Pathologies identified included new or worsened valvular disease (six patients), new or worsened pulmonary hypertension (five patients), a significant decline in ejection fraction (one patient), a newly identified left ventricular outflow tract obstruction (one patient), and a new diagnosis of hypertrophic cardiomyopathy (one patient).

The indications or reasons for TTE given in the orders included clinical concern regarding worsening heart failure (29%), a new or worsening murmur (25%), no clear indication (e.g., “pre-operative clearance” or “evaluate LV function”; 29%), or other cardiac clinical concern (e.g., “new-onset atrial fibrillation” or “elevated troponin levels”; 17%).

When the performance of the CPGs was compared (Table 3), TTE was conducted in accordance with the respective criteria 32% to 66% of the time. The sensitivity of the CPGs ranged from 71% (ESC/ESA and AAGBI) to 100% (ACC/AHA and SIGN). The CPGs’ specificity ranged from 37% (BSE) to 74% (ESC/ESA).

**Table 3:** Performance (sensitivity and specificity) of the five CPGs

	Guideline				
	ACC/AHA	BSE	ESC/ESA	AAGBI	SIGN
TTEs performed in accordance with guidelines	66%	65%	32%	50%	66%
Sensitivity	100%	79%	71%	71%	100%
Specificity	40%	37%	74%	54%	40%
Reduction in TTE^a^	34%	35%	68%	50%	34%
Missed pathology^b^	0%	12%	3%	4%	0%

*CPG* clinical practice guideline, *ACC/AHA* American College of Cardiology/American Heart Association*, BSE* British Society of Echocardiography, *ESC/ESA* European Society of Cardiology/European Society of Anaesthesiology, *AAGBI* Association of Anaesthetists of Great Britain and Ireland, *SIGN* Scottish Intercollegiate Guidelines Network, *TTE* transthoracic echocardiography

^a^Potential percentage reduction in TTE ordering if CPGs were followed

^b^Percentage of patients with pathology detected by TTE that would have been missed if CPGs were followed

## Discussion

The pre-operative management of elderly patients with osteoporotic hip fractures is challenging. In the context of evolving treatment options and evidence, CPGs can provide expert-reviewed, evidence-based guidelines to help design treatment pathways for these medically frail patients. The optimal guidelines would help minimize unnecessary testing while still identifying patients who could have a significant pathology that would be seen on TTE and that might change peri-operative management. In our series, new information with the potential to directly alter management was found in 14% of 100 patients undergoing TTE, but the indications for undergoing TTE were not always clear in the first place. This highlights the importance of having guidelines with criteria that clearly spell out when to administer resource-intensive tests such as TTE.

This analysis has several limitations. First, the study is retrospective, and a well-established protocol for determining which patients required TTE was not applied in this patient group. As a result, our findings are heavily dependent on our institution’s protocol, which may affect generalizability. Also, the retrospective nature of the study necessitated reliance on documentation of the medical team findings and the indications for TTE, which could affect the accuracy of our analysis of the pre-operative assessment. Similarly, we were only able to screen the study reports for information that had the potential to change management, rather than any concrete management changes that actually occurred. Although this may limit the specificity of the results, we believe this study provides valuable data supporting the argument that TTE performed outside of standard CPG recommendations rarely provides new information that alters peri-operative management decisions.

This analysis shows that these CPGs (some of which were initially developed for elective surgery) have different levels of performance when applied to the pre-operative evaluation of patients with osteoporotic hip fracture. The guidelines had sensitivities ranging from 71 to 100%, with specificities ranging from 37 to 74%. If incorporated into practice, the highest-performing CPGs (ACC/AHA, ESC/ESA, and SIGN) could translate into a 30 to 60% reduction in TTE use, while keeping the rate of missed pathology to 3% or lower, depending on the guideline chosen. In this patient cohort, the most sensitive guidelines tended to focus on a change in clinical status in patients with known disease or clinical concern regarding new-onset disease of at least moderate severity, reinforcing the importance of accurate history taking and physical examination when screening patients pre-operatively.

In conclusion, our study demonstrates that established CPGs have variable performance when it comes to determining a need for echocardiography in patients with osteoporotic hip fractures. CPGs that focus on worsening symptoms or clinical examination findings that raise concerns regarding moderate or severe disease (ACC/AHA, SIGN) had the highest sensitivity and may be the most suitable for use in screening. Our hope is that thoughtful application of established guidelines to determine the need for TTE can minimize unnecessary delays in surgery and lessen associated resource use while still identifying patients at risk.

## Electronic supplementary material

ESM 1 (PDF 1225 kb)

ESM 2 (PDF 1224 kb)

ESM 3 (PDF 1224 kb)

ESM 4 (PDF 1224 kb)
